# *Bertiella studeri* Infection in Children, Sri Lanka

**DOI:** 10.3201/eid2608.200324

**Published:** 2020-08

**Authors:** Anjalie Amarasinghe, Thanh H. Le, Susiji Wickramasinghe

**Affiliations:** University of Peradeniya, Peradeniya, Sri Lanka (A. Amarasinghe, S. Wickramasinghe);; Vietnam Academy of Science and Technology, Hanoi, Vietnam (T.H. Le)

**Keywords:** *Bertiella studeri*, zoonotic infection, pediatric patients, mitochondrial markers, NAD1, COX1, nuclear ribosomal DNA markers, ITS2, 28S, 18S, phylogeny, zoonoses, parasites, Sri Lanka

## Abstract

We provide a detailed molecular and phylogenetic description of *Bertiella studeri* tapeworms infecting children in Sri Lanka. Our findings can be used to identify multiple species of *Bertiella* tapeworms that can infect human hosts in the Old World.

The genus *Bertiella*, which has 29 known tapeworm species, belongs to the subfamily *Anoplocephalinae* of the *Anoplocephalidae* family (*1*). These tapeworms are common parasites in the small intestine of primates (*2*). Of these species, only *B. studeri*, *B. mucronata*, and *B. satyri* (*3*), which was recently redescribed as a different species (*4*), can infect humans (*4*,*5*). Children acquire this infection usually by eating contaminated fruits or by ingesting contaminated soil. The earliest identified cases of human bertiellosis in Sri Lanka occurred in 1975; these cases and 1 further case were reported in 1976. Six cases were reported in the literature from 1988–2006 (*6*). The most recent report was in 2006 from Rathnapura, Sabaragamuwa Province, Sri Lanka (*7*). 

The morphologic, taxonomic, and molecular analysis of several species classified in the family *Anoplocephalidae* are not well documented (*6*). A recent study has identified an unexpected genetic diversity that suggests the existence of several *Bertiella* species in primates and humans (*6*,*8*)*.* Multiple species of *Bertiella* tapeworms may infect humans in the New World and the Old World. It is not certain whether the Old World and New World *Bertiella* infections, previously all identified as *B. studeri* or *B. mucronata*, actually represent multiple different species; the true taxonomic distinction and geographic distribution of these 2 species are not entirely clear (*6*). Furthermore, diagnosis entirely based on egg morphology, size, and geographic distribution is insufficient to discriminate *B. studeri* tapeworms from other *Bertiella* spp. infecting humans (*9*). 

This study provides the molecular analysis of the *B. studeri* tapeworms infecting children in Sri Lanka and describes phylogenetic relationships for this species. The Ethics Review Committee in the Faculty of Medicine, University of Peradeniya, Sri Lanka approved this study (protocol no. 2019/EC/03).

## The Study

We conducted a retrospective study using tapeworm proglottids (Appendix Figure 1) from 24 pediatric patients referred to the Department of Parasitology, Faculty of Medicine, University of Peradeniya, Peradeniya, Sri Lanka, during 2007–2017. Patients were all <10 years of age (range 3.5–9 years). No other epidemiologic data were available. 

We extracted genomic DNA separately using a commercial DNA extraction kit (PureLink; Invitrogen, https://www.thermofisher.com). We amplified 2 mitochondrial markers, nicotinamide adenine dinucleotide hydrogenase subunit 1 gene (NAD1) and cytochrome c oxidase subunit 1 gene (COX1), and 3 nuclear ribosomal markers, the second internal transcribed spacer region (ITS2), 28S large subunit ribosomal region (28S), and 18S rRNA gene (18S), using the specified primers and PCR conditions (Appendix Table). We subjected the PCR products to Sanger sequencing; only the ethanol-preserved samples provided a sufficient amount of DNA for sequencing (Appendix). We inferred molecular phylogenetic analysis and evolutionary history using maximum-likelihood and Bayesian methods.

**Figure 1:**
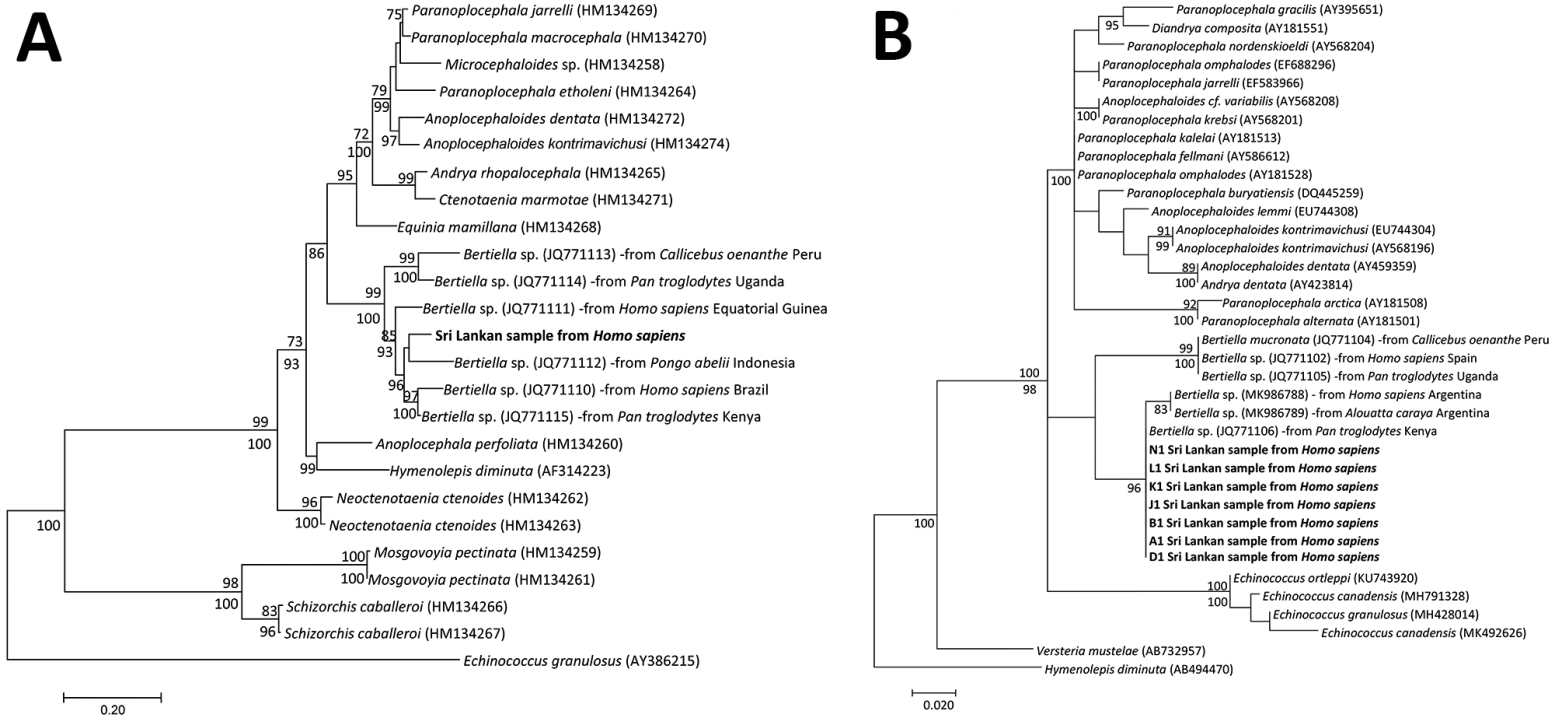
Molecular phylogeny of the mitochondrial markers in a study of *Bertiella* tapeworms in children in Sri Lanka. Bold text indicates *B.* *studeri* samples from Sri Lanka. A) Maximum-likelihood tree containing 25 taxa constructed by the analysis of partial NAD1 sequence alignment. B) Maximum-likelihood tree containing 37 taxa was constructed by the analysis of partial COX1 sequence alignment. Numbers above the nodes indicate the percentages of 1,000 nonparametric bootstrap pseudoreplicates (>70) and below the nodes the percentages of 1,000 Bayesian posterior probabilities (>70). GenBank accession numbers are provided for reference sequences. Scale bars represent nucleotide divergence.

Phylogenetic analysis identified a monophyletic group of *Bertiella* species in all 5 maximum-likelihood trees. The NAD1 region revealed several clades within the *Bertiella* monophyletic group (Figure 1, panel A); sequence similarity search identified 90.19% match with *Bertiella* species (GenBank accession no. JQ771111). The COX1 sequence similarity search identified 95.10% match with *Bertiella* species (GenBank accession no. JQ771106); COX1 analysis identified 2 clades in the *Bertiella* monophyletic group (Figure 1, panel B). *Bertiella* species from human hosts, acquired in Equatorial Guinea and Argentina, and *B. mucronata* (New World) from *Callicebus oenanthe* monkeys were separated from the Sri Lanka clade (Figure 1, panel B). 

**Figure 2:**
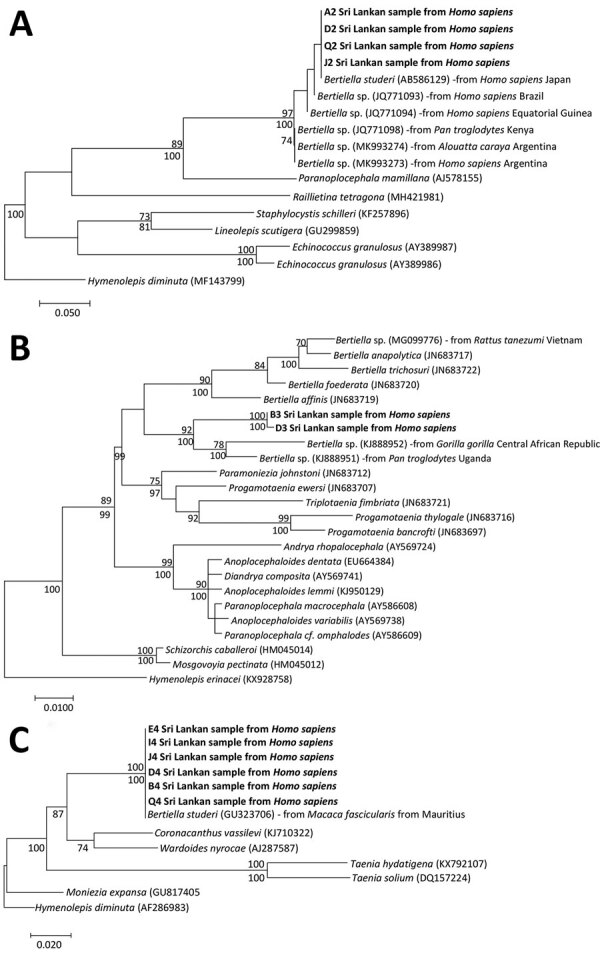
Molecular phylogeny of the nuclear ribosomal markers in study of *Bertiella* tapeworms in children in Sri Lanka. Bold text indicates *Bertiella* *studeri* samples from Sri Lanka. A) Maximum-likelihood tree containing 17 taxa, constructed by the analysis of partial ITS2 sequence alignment. B) Maximum likelihood tree containing 24 taxa, constructed by the analysis of partial 28S sequence alignment. C) Maximum-likelihood tree containing 13 taxa, constructed by the analysis of partial 18S sequence alignment. Numbers above the nodes indicate the percentages of 1,000 nonparametric bootstrap pseudoreplicates (>70) and below the nodes the percentages of 1,000 Bayesian posterior probabilities (>70). GenBank accession numbers are provided for reference sequences. Scale bars represent nucleotide divergence.

The ITS2 sequences showed 99.35% similarity with *B. studeri* (GenBank accession no. AB586129) and 100% similarity with *Bertiella* species (GenBank accession no. JQ771096). All the *B. studeri* sequences from Asia are in 1 clade. The second clade included 3 sequences from *Pan troglodytes* chimpanzee in Kenya, 1 from a human infection acquired in Equatorial Guinea, and 1 from a human host in Brazil (Figure 2, panel A). The 28S rRNA gene analysis revealed 2 clades for *Bertiella* from humans and nonhuman primates (Figure 2, panel B). The sequence similarity search revealed 94.66% similarity with *Bertiella* species (GenBank accession no. KJ888951). Furthermore, we identified a single-nucleotide polymorphism in 28S rRNA region (T to C) between the samples from Sri Lanka that suggest genetic diversity (Appendix Figure 2, panel A). In the ML tree for 18S rRNA region, Sri Lanka samples and *B. studeri* obtained from *Macaca fascicularis* macaque formed a single clade (Figure 2, panel C). The sequence similarity search for 18S rRNA region identified 99.84% match with *B. studeri* (GenBank accession no. GU323706). Furthermore, 18S rRNA region in the Sri Lanka samples have a single-nucleotide polymorphism (T to C) with the *Bertiella* sequence from *M. fascicularis* (Appendix Figure 2, panel B). 

Records we examined showed patients had white, flat, motile worm segments in stools, and some patients had reported abdominal disturbances and intermittent diarrhea. Previous studies reported recurrent abdominal pain and continuous perianal itching, anorexia, weight loss, and intermittent diarrhea in infected patients (*6*); however, these symptoms are not unique to *Bertiella* infection, and so the correct diagnosis of bertiellosis is important. Treatment failure for *B. studeri* worms using niclosamide was reported in a 30-month-old patient in Sri Lanka in 2004 (*10*) and in a 5-year-old patient in Sabaragamuwa Province, Sri Lanka (*7*). 

## Conclusions

Our results suggest an intraspecific diversity of *Bertiella* tapeworms*.* Such diversity may occur according to the host and the geographic location. A previous study conducted by Doležalová et al. (*8*,*11*) has suggested a broad genetic diversity among the *Bertiella* species in primates and humans; further studies are required to support this suggestion. According to the available demographic data, most of the patients resided in Central Province, Sri Lanka; the most likely reason that they comprised most patients is the *Bertiella* tapeworm reservoir hosts, particularly Ceylon torque monkey (*Macaca sinica*) and gray langur (*Presbytis entellus*), that inhabit this region (*12*,*13*). Over time, these monkey populations have lost their habitats due to deforestation and rapid urbanization in Sri Lanka; they are now regular visitors in suburban and urban areas scavenging for food near human settlements, which has increased human exposure to *B. studeri* infection (*14*,*15*). 

Unavailability of molecular data for *B. studeri* 28S, COX1, and NAD1 markers in GenBank was a constraint that we encountered during phylogenetic analysis. In our study, we generated molecular data for 2 mitochondrial markers (NAD1, COX1), and 3 nuclear ribosomal markers (28S, 18S, ITS2) and submitted them to GenBank (Appendix). The molecular data obtained can be used for further analysis in diagnostics, to discern phylogenetic relationships and evolutionary correlations, and to understand the transmission dynamics of *B. studeri* tapeworms. Our data may also be used to assist in elucidating if multiple species of *Bertiella* sp. tapeworms infect human hosts in the Old World.

Appendix Additional information about *Bertiella studeri* infection in children, Sri Lanka.
