# Supplementary material for: Bertiella studeri Infection in Children, Sri Lanka
Source: Emerg Infect Dis. 2020 Aug;26(8):1889–92. doi: 10.3201/eid2608.200324 (PMC7392411; doi:10.3201/eid2608.200324)
Supplement: Appendix — Additional information about Bertiella studeri infection in children, Sri Lanka. [file 20-0324-Techapp-s1.pdf]

Article DOI: https://doi.org/10.3201/eid2608.200324

Bertiella studeri Infection in Children, Sri Lanka

Appendix

PCR Conditions and Concentrations

We analyzed nicotinamide adenine dinucleotide hydrogenase subunit 1 gene (NAD1), cytochrome c oxidase subunit 1 gene (COX1), and 3 nuclear ribosomal markers, the second internal transcribed spacer region (ITS2), 28S large subunit ribosomal region (28S), and 18S rRNA gene (18S). For all 5 markers, we conducted PCR in a final volume of 25 µL containing 4 µL 25mM MgCl2, 2 µL of 2.5 mM dNTPs, 2.5 µL of 10X PCR buffer, 0.5 µL of 5U/µL Taq DNA polymerase, 10 pmol µL each primer, and 5 µL of template DNA. For NAD1, the PCR conditions were used as in Littlewood et al., 2008 (*1*). For COX1, ITS2, 28S, and 18S, the PCR conditions were as follows: initial denaturation at 94°C for 5 min, 30 cycles of denaturation at 94°C for 30 s, annealing at 55°C for 30 s, extension at 72°C for 1 min, and the final extension at 72°C for 5 min. Then, PCR products were examined by running 5 µL of each product in a 1.5% agarose gel and subsequently stained with Diamond Nucleic Acid Dye (Promega, https://www.promega.com).

Sequence Annotation

Of the 24 samples, 22 gave positive results for at least 1 primer set. In this study, only the ethanol-preserved samples gave a sufficient amount of DNA to carry out the sequencing. Previous studies have shown that formalin preservation makes the tapeworm tissues unsuitable for DNA extraction *(6,7)*. Sequence editing was carried out manually using BioEdit version 7.0.5.3 (*8*). The sequence similarity search was done using NCBI BLAST (https://blast.ncbi.nlm.nih.gov/Blast.cgi). Multiple sequence alignments were obtained using ClustalW version 2.0 (https://www.genome.jp/tools-bin/clustalw).

Sequence Analysis

We analyzed NAD1 sequence alignment and constructed a maximum-likelihood tree of the mitochondrial markers (Figure 1). The nucleotide sequence obtained for NAD1 was 891 bp

long. The translated NAD sequence contained 296 aa and was submitted to the GenBank (accession no. MN 982427). COX1 sequences were submitted to GenBank under accession nos. MN982420, MN982421, MN982422, MN982423, MN982424, MN982425, MN982426). Lengths of these sequences were 387 bp, 393 bp, 386 bp, 386 bp, 394 bp, 393 bp, and 394 bp respectively.

Translated sequences in ExPASy (https://web.expasy.org/translate) contained 124 aa and all 7 sequences were similar to each other. Analysis of both mitochondrial markers was conducted by ML method in MEGA version 7.0.26 (https://www.megasoftware.net). Bayesian analysis was implemented in MrBayes (https://nbisweden.github.io/MrBayes), with the model GTR. Four Markov Chain Monte Carlo chains were run for 1 million generations. They were applied as 3 heated chains and 1 cold chain. The four chains reached burn-in time by 200,000 generations. The frequency of clades in trees was sampled for every 100 generations. Both ML and Bayesian trees had the same topologies.

We conducted molecular phylogeny of the nuclear ribosomal markers (Figure 2). In analysis of partial ITS2 sequence alignment, the sequences obtained did not show any nucleotide variance. We submitted sequences to GenBank (accession nos. MN982881, MN982882, MN982883, and MN982884); lengths were 240 bp, 221 bp, 213, bp, and 219 bp respectively.

Analysis of partial 28S sequence alignment yielded 2 sequences, the lengths of which were 789 bp and 791 bp. Sequences were deposited into GenBank (accession nos. MN982722–MN982723. C) Analysis of partial 18S sequence alignment yielded 4 sequences, the lengths of which were 642 bp, 642 bp, 642 bp, and 620 bp; sequences were deposited into GenBank (accession nos. MN982715, MN982716, MN982717, MN982718, MN982719, and MN982720.

We analyzed 3 nuclear ribosomal markers by maximum-likelihood using MEGA version 7.0.26 (https://www.megasoftware.net). Furthermore, we conducted Bayesian analysis in MrBayes (https://nbisweden.github.io/MrBayes/index.html), with the model general time reversible plus gamma 4 plus invariate sites. Four Markov Chain Monte Carlo chains were run for 1 million generations, applied as 3 heated chains and 1 cold chain. The 4 chains reached burn-in time by 250,000 generations. The frequency of clades in trees was sampled for every 100 generations. The maximum-likelihood and Bayesian trees had the same topologies.

References

1. Littlewood DT, Waeschenbach A, Nikolov PN. In search of mitochondrial markers for resolving the phylogeny of cyclophyllidean tapeworms (Platyhelminthes, Cestoda) –a test study with Davaineidae. Acta Parasitol. 2008;53:133–44. https://doi.org/10.2478/s11686-008-0029-4

2. Bowles J, McManus DP. Genetic characterization of the Asian *Taenia*, a newly described taeniid cestode of humans. Am J Trop Med Hyg. 1994;50:33–44. PubMed https://doi.org/10.4269/ajtmh.1994.50.1.TM0500010033

3. Littlewood DT, Johnston DA. Molecular phylogenetics of the four *Schistosoma* species groups determined with partial 28S ribosomal RNA gene sequences. Parasitology. 1995;111:167–75. PubMed https://doi.org/10.1017/S003118200006491X

4. Bowles J, Blair D, McManus DP. A molecular phylogeny of the human schistosomes. Mol Phylogenet Evol. 1995;4:103–9. PubMed https://doi.org/10.1006/mpev.1995.1011

5. Doležalová J, Vallo P, Petrželková KJ, Foitová I, Nurcahyo W, Mudakikwa A, et al. Molecular phylogeny of anoplocephalid tapeworms (Cestoda: Anoplocephalidae) infecting humans and non-human primates. Parasitology. 2015;142:1278–89. PubMed https://doi.org/10.1017/S003118201500058X

6. Taleb-Hossenkhan N, Bhagwant S. Molecular characterization of the parasitic tapeworm *Bertiella studeri* from the island of Mauritius. Parasitol Res. 2012;110:759–68. PubMed https://doi.org/10.1007/s00436-011-2550-x

7. Taleb-Hossenkhan N, Bhagwant S, Gourrege N. Extraction of nucleic acids from ancient formalin- and ethanol-preserved specimens of the tapeworm *Bertiella studeri*: which method works best? J Parasitol. 2013;99:410–6. PubMed https://doi.org/10.1645/GE-3197.1

8. Hall T, Biosciences I, Carlsbad C. BioEdit: an important software for molecular biology. GERF Bull Biosci. 2011;2:60–1.

Appendix Table. Amplified regions and the primers used for the PCR amplification in analysis of *Bertiella* sp. tapeworms in children. Sri Lanka

Amplified region	Primer name*	Sequence (5′-3′)	Size of the amplicon, bp	Reference
NAD1	Cyclo-Nad1F (f)<br>Cyclo-trnNR (r)	GGN TAT TST CAR TNT CGT AAG GG<br>TTC YTG AAG TTA ACA GCA TCA	730–900	(*1*)
COX1	JB3 (f)<br>JB4.5 (r)	TTT TTT GGG CAT CCT GAG GTT TAT<br>TAA AGA AAG AAC ATA ATG AAA ATG	393	(*2*)
28S	LSU5 (f)<br>LSU5 (r)	ACC CGC TGA ATT TAA GCA<br>TCC TGA GGG AAA CTT CGG	794	(*3*)
18S	BF (f)<br>BR (r)	GGA CAC TAT GAG GAT TGA CAG A<br>CCT TTC GGG GCA CCA AGA TGG	600	(*4*)
ITS-2	3S (f)<br>28A (r)	CGG TGG ATC ACT CGG CTC GT<br>CCT GGT TAG TTT CTT TTC CTC CGC	598 and 664	(*5*)

***f**, forward; r, reverse.

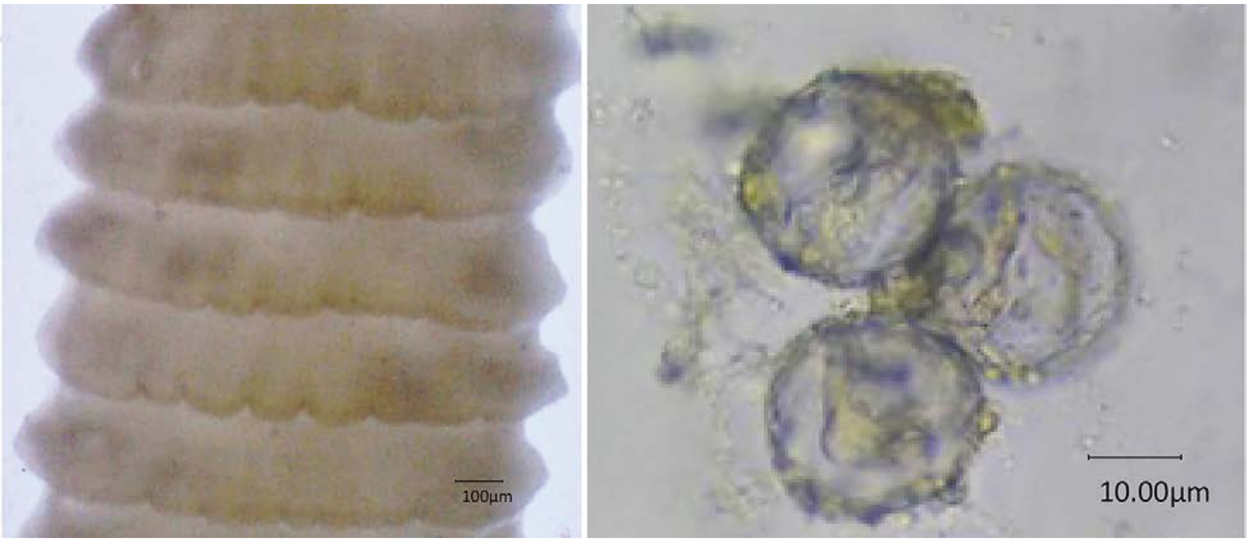

Appendix Figure 1. Morphological characteristics observed during the study of *Bertiella* spp. in children, Sri Lanka. A) *Bertiella studeri* proglottids. B) *B. studeri* eggs liberated from the proglottids.

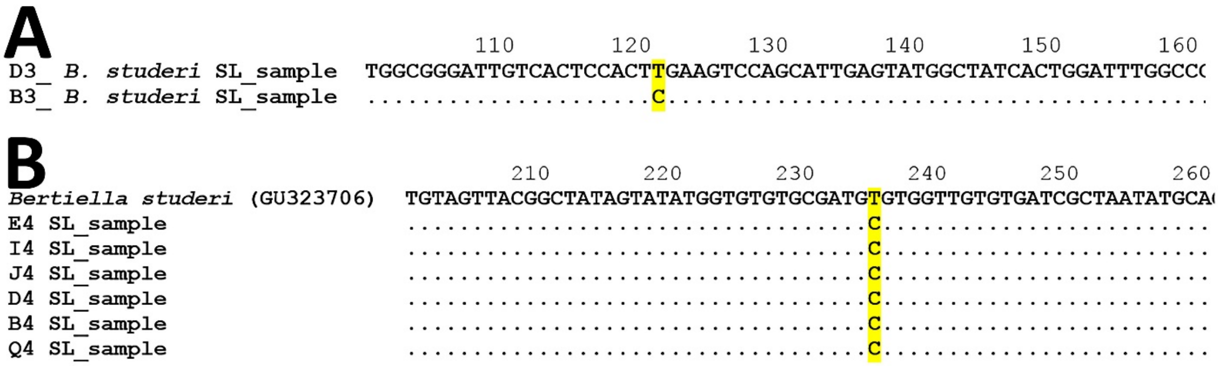

Appendix Figure 2. Nucleotide sequence alignments showing SNPs in the 28S and 18S regions. A) alignment of the 2 sequences obtained for the 28S region. B) Alignment of the 6 sequences of 18S region Sri Lankan (SL) samples with *B. studeri* reference sequence (GenBank accession no. GU323706). Highlighting indicates SNPs in each alignment. Dots indicate identity with the base in the top line.
